# Freeze Tolerance in Sculpins (Pisces; Cottoidea) Inhabiting North Pacific and Arctic Oceans: Antifreeze Activity and Gene Sequences of the Antifreeze Protein

**DOI:** 10.3390/biom9040139

**Published:** 2019-04-06

**Authors:** Aya Yamazaki, Yoshiyuki Nishimiya, Sakae Tsuda, Koji Togashi, Hiroyuki Munehara

**Affiliations:** 1 Nanae Fresh-Water Station, Field Science Center for Northern Biosphere, Hokkaido University, Nanae Town Kameda-gun 041-1105, Japan; 2 Bioproduction Research Institute, National Institute of Advanced Industrial Science and Technology, Sapporo 062-8517, Japan; y.nishimiya@aist.go.jp (Y.N.); s.tsuda@aist.go.jp (S.T.); 3 Graduate School of Environmental Sciences, Hokkaido University, Sapporo 060-0810, Japan; minivan.bmc.minivan@gmail.com; 4 Usujiri Fisheries Station, Field Science Center of Northern Biosphere, Hokkaido University, Hakodate 041-1613, Japan; hm@fsc.hokudai.ac.jp

**Keywords:** antifreeze proteins, cold adaptations, Cottoidea, thermal hysteresis

## Abstract

Many marine species inhabiting icy seawater produce antifreeze proteins (AFPs) to prevent their body fluids from freezing. The sculpin species of the superfamily Cottoidea are widely found from the Arctic to southern hemisphere, some of which are known to express AFP. Here we clarified DNA sequence encoding type I AFP for 3 species of 2 families (Cottidae and Agonidae) belonging to Cottoidea. We also examined antifreeze activity for 3 families and 32 species of Cottoidea (Cottidae, Agonidae, and Rhamphocottidae). These fishes were collected in 2013–2015 from the Arctic Ocean, Alaska, Japan. We could identify 8 distinct DNA sequences exhibiting a high similarity to those reported for *Myoxocephalus* species, suggesting that Cottidae and Agonidae share the same DNA sequence encoding type I AFP. Among the 3 families, Rhamphocottidae that experience a warm current did not show antifreeze activity. The species inhabiting the Arctic Ocean and Northern Japan that often covered with ice floe showed high activity, while those inhabiting Alaska, Southern Japan with a warm current showed low/no activity. These results suggest that Cottoidea acquires type I AFP gene before dividing into Cottidae and Agonidae, and have adapted to each location with optimal antifreeze activity level.

## 1. Introduction

How creatures expand their distribution range by acquiring adaptive traits, and which mechanisms and/or adaptations decide this distribution range is a central and universal problem in evolutionary ecology. Acquisition of thermal tolerance is important for adaptation to cold and hot habitats. For these adaptations, many species express heat shock proteins [1,2], antifreeze proteins (AFPs) and antifreeze glycoproteins (AFGPs) [3,4].

Many marine species inhabiting icy seawater produce AF(G)Ps to prevent their body fluids from freezing [3,5,6], and type I–III AFPs and AFGPs have been found in marine fishes [7,8,9,10,11]. These proteins are thought to inhibit ice growth by irreversible adsorption to the surface of nascent ice crystals generated at the freezing point of water [12]. The acquisitions of AF(G)P enable adaptation to cold environments, and are closely related to geographic events. The cold period started after 35 million years ago (Ma) when the Antarctica was formed [13], and AFGP acquisition enabled Notothenia fishes survive these cold periods. These fishes rapidly speciated and adaptively radiated, because fishes without cold tolerance leave Antarctica during cold periods. On the other hand, in the Arctic Ocean, the formation of ice-sheets began 47.5 Ma [14], and developed after 3 Ma. The genus *Myoxocephalus*, inhabiting the northern hemisphere, included in the Cottidae, are estimated to have acquired AFP at least 30 Ma [15]. In addition, cunner is thought to have acquired AFP at the last glaciation, because this species express AFPs only in epithelial cells [16].

Superfamily Cottoidea was recently reconstructed with 7 families, 94 genera and 387 species; Jordanidae, Rhamphocottidae (Ereuniidae), Scorpaenichthyidae, Agonidae (Hemitripteridae), Cottidae, Psychrolutidae, and Bathylutichthyidae [17]. The species are mainly distributed in the high latitude area of the northern hemisphere, and 6 families, 22 genera and 72 species are found in the Arctic Ocean [18]. In the superfamily Cottoidea, type II AFPs are found in the family Agonidae (Hemitripterus americanus, Brachyopsis rostratus) [19,20], and a type I AFP is found in the family Cottidae (*Myoxocephalus* spp.) [21]. In the genus *Myoxocephalus*, Arctic species, *M. scorpius*, *M. aenaeus*, and *M. polyacanthocephalus*, show higher antifreeze activity, and subarctic–temperate species, *M. stelleri*, shows lower antifreeze activity [15,21,22]. The deep-sea species *M. octodecemspinosus* express AFP only in the skin [23], not in plasma [24]. A total of 5 genera and 5 species possess antifreeze activity in the superfamily Cottoidea: *Hemilepidotus jordani*, *Gymnocanthus herzensteini*, *Furcina osimae* [25,26], longsnout poacher, *Brachyopsis rostratus* [27], and sea raven, *Hemitripterus americanus* [9]. However, the antifreeze activity in almost species is still unknown in this superfamily. To understand cold adaptation in the superfamily Cottoidea, we clarified DNA sequences encoding type I AFP for 2 families and 3 species, and antifreeze activities for 4 families and 32 species. We lastly discussed about the acquisition of AFP and adaptation to the cold region in the superfamily Cottoidea.

## 2. Materials and Methods

### 2.1. Sample Collections

**Figure 1:**
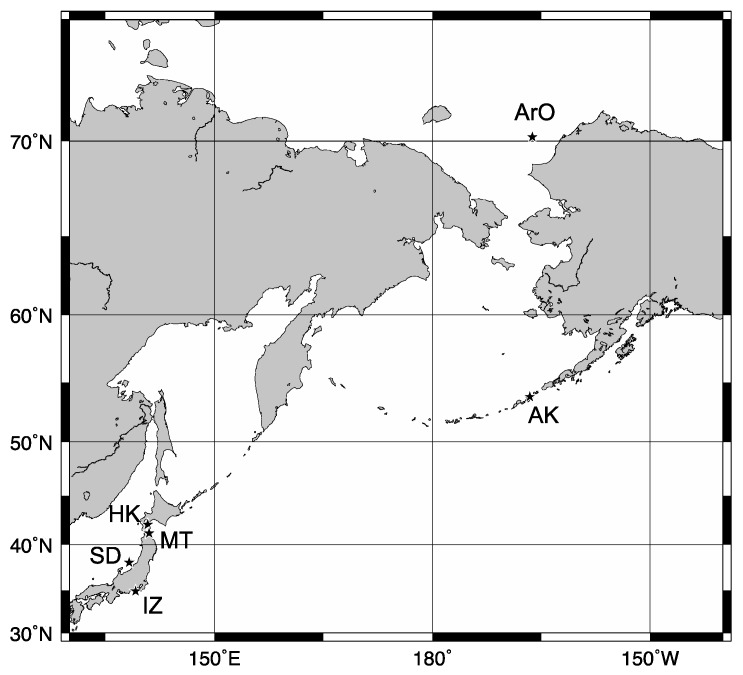
Sampling locations are represented with stars. ArO: the Arctic Ocean, AK: Alaska, HK: Hokkaido, MT: Mutsu, SD: Sado Island, IZ: Izu.

Samples were collected from the Arctic Ocean, Alaska, Hokkaido, Mutsu, Sado Island and Izu (Figure 1). Detailed information of each location is presented in Table 1. Sculpins were identified by morphological characters using the “Fishes of Alaska” [28], “Fishes of Japan” [29] at laboratory. Muscle tissues of five species from the Arctic Ocean (166.2308333° E, 70.18950° N), nine from Alaska (166.5858000° E, 53.8383333° N), thirteen from Hokkaido (140.8086° W, 42.07233333° N), three from Mutsu bay (140.9923333° W, 41.191° N), five from Sado Island (138.241° W, 38.07183333° N), and one from Izu (139.1303° W, 34.8847° N) were collected for antifreeze activity measurements (Table 1). Since the skin portion contains a mucoid substance that prevents the activity measurements, we removed that portion from the sample as much as possible. This removal also minimized the contamination of the skin-type AFP. These muscle tissues were preserved at −25 °C until measurement. For the determination of AFP sequences, the fresh dorsal fins and livers from *Hemilepidotus jordani* and *Gymnocanthus tricuspis*, collected in Alaska, and *Porocottus allisi*, collected in Hokkaido, were stocked overnight at 4 °C in RNA*later*^®^ Stabilization Solution (Life Technologies^TM^, Carlsbad, CA, USA) to thoroughly penetrate cells, and then stocked at −80 °C for long storage. These treatments were according to the Hokkaido University Regulations of Animal Experimentation.

### 2.2. Measurement of Antifreeze Activities

DeVries [30] showed that the freezing point of blood that does not contain AFP is 0.01 °C. Several articles have shown that type I–III AFP were successfully purified from fish muscle homogenates, and described fish muscle as a rich source of AFP [20,25,31,32,33]. It has also been shown that the ice-shaping ability of AFP is not nullified by contaminants present in body fluid, such as blood serum. Hence, we used minced muscle homogenates as a source material for examining antifreeze activity. After weighing the muscle tissues, they were homogenized, using a Beadbeater (Biospec, Bartlesville, OK, USA), in tubes containing an equal amount of distilled water, and centrifuged at 15,000 rpm for 15 minutes to obtain supernatant solutions for antifreeze activity measurement. Antifreeze activity was measured using a temperature-controlled stage, as described by Takamichi et al. [34]. The supernatant solutions were super-cooled −55 °C/min, heated up 5 °C/min after being complete frozen, and cooled down −1 °C/min. As ice crystals change shapes, depending on the AFP concentrations or the ice-binding power [35,36,37], the level of antifreeze activity was defined as follows: “high level” with the formation of bi-pyramidal ice crystals, “low level” with hexagonal ice crystals, and “zero” with other shapes.

### 2.3. cDNA Syntheses, Cloning, and Sequencing

Total RNA was extracted from fin clips that collected at Alaska and Hokkaido using the QuickGene RNA tissue kit S II (QuickGene 800, Kurabo, Japan) and DNase I Amplification Grade (Invitrogen^TM^, Carlsbad, CA, USA) following the manufacturer’s instructions. The first strand cDNA was synthesized from the RNA, using SuperScript VILO (Invitrogen^TM^, Carlsbad, CA, USA) according to the manufacturer’s instructions.

Nested polymerase chain reaction (PCR) was conducted to select candidate AFP genes from each reverse transcription (RT)-PCR amplicon. Five primers (5’1, 5’2, Deg1, 3’1 and 3’2) [38] and one primer (LS-ST-P2) [15] were used in three combinations (5’1 and 3’1, 5’2 and 3’1, and LS-ST-P2 and 3’1) in the first PCR, and then the same four primers except 5’1 and 3’1 primers in all possible combinations were used for the second PCR, with the first PCR products serving as the DNA templates (Table 2). The concentration of each primer was 0.4 µM. KOD-Plus- Ver. 2 (Toyobo, Osaka, Japan), including KOD DNA polymerase, was used for both PCRs. PCR products from a single RT-PCR amplicon were pooled and sub-cloned, using the TArget Clone^TM^ -Plus- system (Toyobo, Osaka, Japan) according to the manufacturer’s instructions. Transformations into *E. coli* (*ECOS*^TM^ Competent *E. coli* JM109, Nippongene, Tokyo, Japan) were conducted by the heat shock method, and bacteria were then incubated on LB/amp/IPTG/X-gal plates containing ampicillin for 12 hours at 37 °C. After blue/white selection, all the white colonies were re-streaked and amplified by colony PCR. Each reaction was conducted in a total volume of 50 µL, which consisted of 0.2 µM of each primer (universal T7 and T3 promoter primers), 1x EmeraldAmp PCR Master Mix (Takara Bio Inc., Siga, Japan), and the picked DNA sample. The PCR conditions were as follows: pre-denaturing at 95 °C for 1 min, 30 cycles of denaturing at 94 °C for 30 s, annealing at 60 °C for 30 s, extension at 72 °C for 3 min, and final extension at 72 °C for 10 min. PCR products were purified using NucleoSpin^®^ Gel and PCR Clean-up according to the manufacturer’s instructions (Macherey-Nagel, Düren, Germany). Sequencing was conducted with two directions using same universal primers (T7 and T3 promoters) using Applied Biosystems 3730x1 DNA analyzer (Life Technologies, Carlsbad, CA, USA) by Macrogen Japan (Kyoto, Japan).

### 2.4. Bioinformatics Analyses

To identify the phylogenetic relationships among Cottoidea, 40 genera and 40 species cited from GenBank were used in these analyses (Table S1). Sequences from four loci (RAG1, COI, Cyt*b*, and 12SrRNA–tRNA^Val^–16SrRNA) were aligned using MEGA6 with default settings and adjusted visually. Gaps were identified and deleted using MAFFT ver. 7 [39] with the E-INS-i option, and trimAl ver. 1.2 [39,40] with no gap option. Kakusan4 [41] was used to determine the appropriate model for each gene. The maximum likelihood method was also explored using RAxML ver. 7.2.8 [42]. The blanches were colored by the Phytools. The northern distributional limit of the species and the distributional depth range were cited from FishBase [43].

## 3. Results

### 3.1. Antifreeze Activity

**Figure 2:**
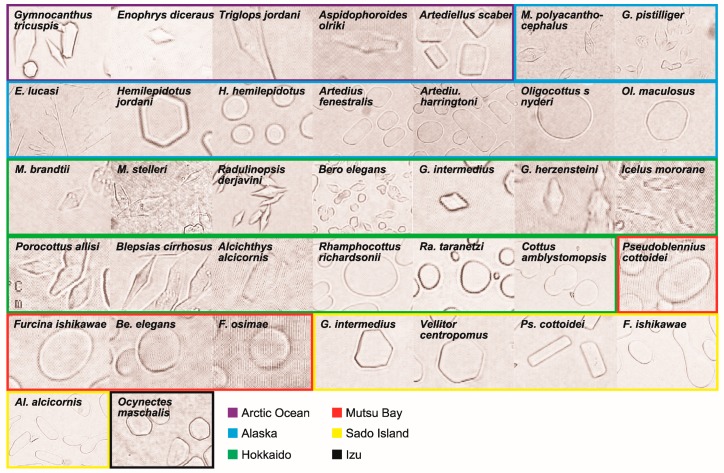
Ice-crystal photos of all species. Bi-pyramidal ice-crystal represents high antifreeze activity, hexagonal ice-crystal represents low antifreeze activity, and other shapes represents inactivity. Solid squares are colored by location; purple, blue, green, red, yellow, and black show the Arctic Ocean, Alaska, Hokkaido, Mutsu Bay, Sado Island, and Izu, respectively.

Fourteen of the 32 species examined showed high antifreeze activity, 5 showed low activity, and 13 showed no activity (Table 1, Figure 2, some species was duplicated due to being collected from several locations, see Table 3). Many species inhabiting the Arctic Ocean showed high-level antifreeze activity, while most of the southern species from Sado and Izu showed no activity.

**Figure 3:**
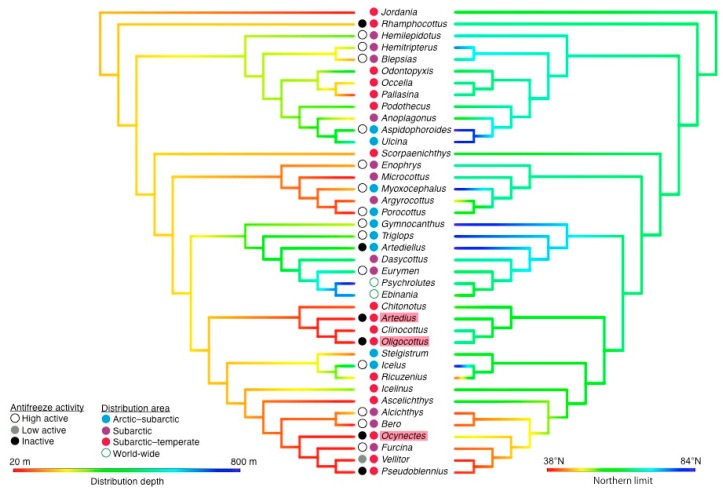
Molecular phylogeny and antifreeze activity in the superfamily Cottoidea. White, grey, and black circles represent genera with high, low, and no antifreeze activity. Colored circles represent the distribution area; blue, purple, and red show the genera inhabiting the Arctic–subarctic, subarctic, subarctic–temperate areas, respectively; the green-lined circles show the genera inhabiting world-wide. The left tree is colored by the distribution depth; 20 m depth is colored red and 800 m depth is colored blue. The right tree is colored by the northern limit of the distribution area; 38° N is colored red and 84° N is colored blue.

In the family Cottidae, 12 species showed high activity, 4 showed low activity and 11 showed no activity. In the family Agonidae, 2 species showed high activity, 1 showed low activity and 1 showed no activity. The family Ramphocottidae showed no activity (Figure 3).

Almost all species in the Arctic Ocean except *Artediellus scaber*, maintained high-level antifreeze activity, even though they were collected in the summer. Some sculpins inhabiting Alaska exhibited marked activity, but the tide-pool species, including *Artedius* and *Oligocottus*, showed no activity. In Hokkaido, *Radulinopsis taranetzi* and *Cottus amblystomopsis* showed inactivity, and the other 11 species showed either high or low activities. Many species exhibiting no activity inhabited Mutsu Bay, Sado Island, and Izu, which are warm regions. Antifreeze activities differed among species within a genus, being noted in seven genera: *Enophrys*, *Hemilepidotus*, *Radulinopsis*, *Gymnocanthus*, *Artedius*, *Icelus*, and *Myoxocephalus*. Two species of *Radulinopsis*, inhabiting the same latitude and depth range, showed different activities. Antifreeze activity also differed by location for *G. intermedius*, *Alcichthys alcicornis* and *Bero elegans*. On the other hand, the temperate species *Vellitor centropomus* in Sado showed low antifreeze activity.

### 3.2. Genetic Structures of Cottidae Antifreeze Proteins

**Figure 4:**
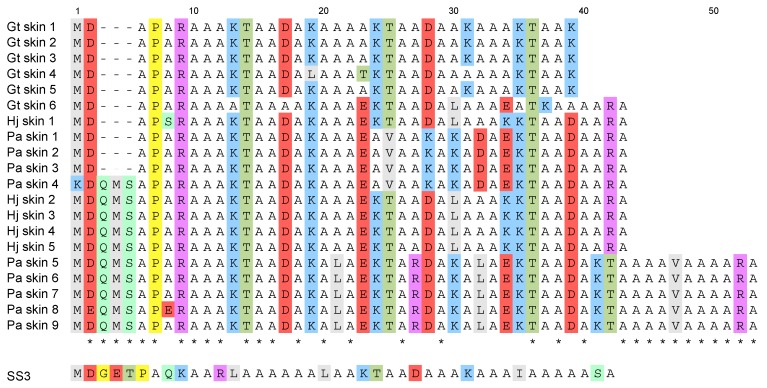
Amino acid sequences of type I antifreeze protein (AFP) from three Cottoidea species, *Gymnocanthus tricuspis*, *Porocottus allisi*, *Myoxocephalus scorpius* (SS3), and *Hemilepidotus jordani*. Thr are colored green. Polar residues (except Thr) are highlighted light green. Basic residues are highlighted blue (Lys) or purple (Arg), acidic are highlighted red (Asp and Glu), hydrophobic residues (except Ala) are highlighted gray (Met, Leu, and Val) and exceptional residues (Pro and Gly) are highlighted yellow. Hyphen (-) represents gaps. Asterisk (*) indicates the conserved site.

A total of 20 nucleotide sequences, encoding a total of 8 distinct AFPs, were identified (Figure 4). The deduced sequences encoded 4 distinct AFPs in *P. allisi*, 2 in *H. jordani* and 3 in *G. tricuspis*. These 8 sequences represent 5 new AFP variants as some of these sequences were previously known from *Myoxocephalus* species. This brings the total number of known sequences from the superfamily Cottoidea to 47. The lengths of AFP nucleotide sequences were 312–359 bp and open reading frame consisted of 40 residues in *H. jordani*, 243–308 bp and 36–40 residues in *G. tricuspis*, and 281–608 bp and 40–53 residues in *P. allisi*. The length and sequences of the 5’- and 3’-untranslated regions (UTR) differed among species (Figure S1). Three species show a preference for a particular Ala codon within the AFP sequences; between 44.0 and 59.0% of the Ala residues were encoded by the GCG codon. Three groups, according to a difference in peptide lengths, were determined as follows: 36-residue short isoform (SI), 40- or 43-residue medium isoform (MI), and 53-residue isoform. The 4 AFP sequences deduced from SI (*G. tricuspis*) are 100% identity to the 36-aa *M. polyacanthocephalus* sequence, one sequence from MI (*P. allisi*) is 100% identity to the 40-aa *M. brandtii* sequence, and nine other sequences from MI (*G. tricuspis*, *P. allisi* and *H. jordani*) are also similar to these sequences. In the coding region, there is 94.2%, 71.2%, and 99.2% identity among SI, MI, and 53-residue isoform, respectively. The 3’-UTRs show highly identity, with 95.3%, 69.6%, and 100% identity within SI, MI, and 53-residue isoform, respectively. The similarity between 53-residue isoform and MI (43-residues) are 79.2% in the coding region and 87.5% in the 3’-UTR. All sequence data were deposited in GenBank (Accession numbers: MK550897–MK550916).

## 4. Discussion

### 4.1. Type I AFP Was Acquired before the Divergence within Cottoidea

The AFP sequences of Porocottus (Cottidae) are similar to those sequences of *Hemilepidotus* (Agonidae), and the AFP sequences of *Gymnocanthus* (Cottidae) are similar to those of *Myoxocephalus* (Cottidae). The 53-residue isoform sequences share more than 90% identity with AFP sequences from the shorthorn sculpin and longhorn sculpin [21,23]. High similarity between Cottidae and Agonidae indicates that the type I AFP is shared within the superfamily Cottoidea. This also suggests that the ancestral gene of type I AFP has been acquired before the divergence within Cottoidea. Moreover, in Agonidae, the longsnout poacher and the sea raven have type II AFPs [9,27], which are categorized into two: Ca^2+^-dependent type II AFP, found in Japanese smelt, *Hypomesus nipponinsis* [32], rainbow smelt, *Osmerus mordax*, and Atlantic herring, *Clupea harengus harengus* [44]; and Ca^2+^-independent type II AFP, found in sea raven, *Hemitripterus americanus* [19], and longsnout poacher, *Brachyosis rostratus* [20]. This Ca^2+^-independent type II AFP is larger than the type I AFPs of sculpins (163 residues in sea raven [45], 133-residues in longsnout poacher [20]). The type II AFPs were acquired by lateral gene transfer [46,47]. In addition, type I AFPs evolved convergence in four families (Pleuronectidae, Cottidae, Liparidae, and Libridae) [38]. Nonetheless, no single family has several AFP types. Our finding was the first report to have two types of AFPs within the family Agonidae. AFP type identification in the remained superfamily Cottoidea has the potential for contributing, not only to our understanding of the cold adaptation process, but also to understanding the unsolved phylogeny within Cottoidea.

### 4.2. Antifreeze Activity and Current Cold Adaptation

Antifreeze activity in Cottoidea was maintained in the Arctic–subarctic area, but declined in the tidal, deep, and temperate regions. Compared with other regions, almost all species inhabiting the Arctic Ocean showed high antifreeze activity. Many species inhabiting Hokkaido also showed high antifreeze activity. In Alaska, Mutsu, and more southern areas in Japan, most species showed low or no antifreeze activity. These differences are affected by sea currents; cold currents in Hokkaido, and warm currents in Alaska, Mutsu and more southern areas. Compared with the northern limit of the species’ distributional area, species located higher than 48° N in the Northwest Pacific Ocean and higher than 66° N in the North Pacific Ocean showed high antifreeze activity, but species located higher than 58° N showed low or no antifreeze activity. Therefore, the antifreeze activity differs even if the species inhabit the same latitude. The grunt sculpin *Rhamphocottus richardosonii*, a member of the family Rhamphocottidae, which showed no antifreeze activity is typically found in temperate regions: Japan, Alaska, and Southern California [43]. This species is thought to have expanded its distribution area along the Aleutian archipelagoes during warm periods, moved southward during cold periods, and then divided into the Northeast and Northwest Pacific Oceans, because many marine species moved to milder environment in the cold periods [48]. Therefore, freeze tolerance is not necessary for this species.

The hamecon, *Artediellus scaber*, inhabiting the Arctic Ocean, which was covered with ice sheet at the time of sampling, did not show antifreeze activity. Although the bottom temperatures were −1.8 °C near the sampling location, there was −0.7 °C at the sampling location. Almost all species in the family Psychlolutidae inhabit deep-water, but one species, the smoothcheek sculpin, *Eurymen gyrinus*, which inhabits shallow-water, shows high antifreeze activity [25]. In the icy-water area, the lumpfish, *Cyclopterus lumpus* (Cyclopteridae), and the sand flounder, *Scophthalmus aquosus* (Pleuronectidae), do not have antifreeze activity in the serum in winter [24]. The longhorn sculpin, which inhabits deep-water, also does not have antifreeze activity in the serum [24], but has antifreeze activity in the skin [23]. The cunner, which recently migrated to the Arctic region, also expresses AFP only in skin and gill filaments [16]. The external epithelia such as skin and gill filaments would be the first tissue to contact with ice. So, the species inhabiting non freeze-risk area and/or recently migrated to the freeze-risk area express AFP only in these tissues. On the other hand, some species of the family Nototheniidae hatch with underdeveloped gill structures, minimizing the risk of ice introduction through these delicate structures [49]. Given together, some hypotheses were proposed: (i) *A. scaber* has AFPs expressed in the muscle and/or serum during winter as known in many species; (ii) *A. scaber* expresses AFPs only in skin; and (iii) *A. scaber* does not have AFPs in the muscle and serum, other physical barriers such as underdeveloped gill structures, elevated osmolality of body fluids and/or mucus on the skin, contribute significantly to the freezing resistance and survival of *A. scaber*.

Many species decline or lose antifreeze activity in deep areas, such as wolffish, snailfish and flounder [24,50,51]. Generally, there is no freezing risk because of the absence of ice nuclei in the deep area. However, in the family Cottidae, some subtidal species maintain antifreeze activity. A lot of species both shallow- and deep-water species in the superfamily Cottoidea spawn in shallow areas during winter and some species lay on the eggs (e.g., *Enophrys bison* [52], *Hemitripterus villosus* [53,54]); so these deep-water species also need freeze tolerance. While subtidal species maintain antifreeze activity, the tide-pool species, such as *Artedius*, *Oligocottus*, and *Ocynectes*, which inhabit Alaska and Izu, do not show antifreeze activity. Tidal areas are hypoxia and dry environments, hence, species inhabit these areas need hypoxia and desiccation tolerance [55]. Alaska and Izu, where inactive species were collected, were not icy during winter. These species might express AFP only in the skin or do not express AFP, due to the expression of genes for hypoxia and desiccation tolerances.

### 4.3. Distributional Expansion to Cold Regions and Acquisition of AFP

Families Cottidae and Agonidae were originated at the Pacific Ocean (Briggs 2003), and almost sculpins and poachers are distributed in the North Pacific Ocean. Since Near, et al. [56] estimated that the superfamily Cottoidea and the family Liparidae diverged about 30 Ma, the Cottoidea is thought to have acquired the ancestral gene of type I AFP after the divergence, when the formation of ice-sheet had already started in the Arctic [14]. The type II AFP is found in only Agonidae in this superfamily, so Agonidae might acquire type II AFP after the divergence of this family.

The six families, 22 genera and 72 species inhabit the Arctic Ocean in the superfamily Cottoidea, and the species with eelpouts (Zoarcidae), which has type III AFP, reach more than half of all of the species in the Arctic Ocean [18]. A few species in other fishes, such as cods (Gadidae) with AFGP, flounders (Pleuronectidae) with type I AFP, salmons and herrings (Salmonidae and Clupeidae) with type II AFP, also inhabit the Arctic Ocean. In Zoarcidae, almost of the species of genus *Lycodes*, found in the Arctic Ocean, are estimated to have speciated about 3 Ma when the ice-sheet had developed [57]. Speciations in the Arctic and/or Atlantic Oceans indicate the adaptive radiations to cold regions occurred, so these fishes could occupy in the Arctic Ocean. Furthermore, the current species in the family Cottidae have hypoxia- and desiccation-tolerance even if the subtidal species [55], and some species adapt to fresh- and brackish-water [58], suggesting that these fishes could survive the past Arctic Ocean than the other fishes when the sea-level and salinity changed by glacial cycles. Given together, the superfamily Cottoidea could occupy the cold regions by acquiring various functional genes such as AFPs.

## 5. Conclusions

We clarified DNA sequence encoding type I AFP for 3 species of 2 families (Cottidae and Agonidae) belonging to Cottoidea. We could identify 8 distinct DNA sequences exhibiting a high similarity to those reported for *Myoxocephalus* species, suggesting that Cottidae and Agonidae share the same DNA sequence encoding type I AFP. Our findings were the first report that the family Agonidae has two types of AFPs. We also examined antifreeze activity for 3 families and 32 species of Cottoidea (Cottidae, Agonidae, and Rhamphocottidae), 14 species showed high antifreeze activity, 5 showed low activity, and 13 showed no activity. Among the 3 families, Rhamphocottidae that experience a warm current did not show antifreeze activity. Antifreeze activities differed among species within a genus (7 genera). Antifreeze activity also differed by location for 3 species. The species inhabiting the Arctic Ocean and northern Japan that often covered with ice floe showed high activity, while those inhabiting Alaska, southern Japan with a warm current showed low/no activity. These results suggest that Cottoidea acquires the ancestral genes of type I AFP before dividing into Cottidae and Agonidae about 30 Ma, and have adapted to each location by expressing optimal antifreeze activity level. Species in Cottoidea could occupy the cold regions by acquiring various functional genes such as AFPs.

## Figures and Tables

**Table 1:** Sampling locations and antifreeze activities. SBT: Sea bottom temperature (°C), *N*: number of species.

Location	Date	SBT	*N*	Antifreeze Activity		
				High	Low	None
Arctic Ocean	July 2013	−0.7	5	4	0	1
Alaska	March 2014	3.6	9	3	1	5
Hokkaido	April 2014	4.4	13	7	3	3
Mutsu Bay	December 2013	10.4	3	0	0	3
Sado Island	February 2014	9.6	5	0	2	3
Izu	March 2015	7.0	1	0	0	1
Summary *			32	14	6	16

* Include duplicated species.

**Table 2:** Primer combinations.

1st Reaction		2nd Reaction	
Forward	Reverse	Forward	Reverse
5’1	3’1	5’2	3’2
		LS-ST-P2	3’2
		Deg1	3’2
5’2	3’1	LS-ST-P2	3’2
		Deg1	3’2
LS-ST-P2	3’1	Deg1	3’2

**Table 3:** Sample information and antifreeze activities in Cottoidea. ArO: Arctic Ocean, AK: Alaska, HK: Hokkaido, MT: Mutsu, SD: Sado Island, IZ: Izu.

Family	Genus	Species	Location	Depth	Date	Antifreeze Activity
Cottidae	*Alcichthys*	*alcicornis*	HK	Subtidal	April 2015	Low
			SD		February 2014	None
	*Artediellus*	*scaber*	ArO	Subtidal	July 2013	None
	*Artedius*	*fenestralis*	AK	Tidal	March 2014	None
		*harringtoni*	AK	Tidal	March 2014	None
	*Bero*	*elegans*	HK	Subtidal	April 2014	Low
			MT		December 2013	None
	*Cottus*	*amblystomopsis*	HK	Stream	April 2014	None
	*Enophrys*	*lucasi*	AK	Subtidal	March 2014	High
		*diceraus*	ArO	Subtidal	July 2013	High
	*Furcina*	*ishikawae*	MT	Subtidal	December 2013	None
			SD		February 2014	None
		*osimae*	MT	Subtidal	December 2013	None
	*Gymnocanthus*	*tricuspis*	ArO	Subtidal	July 2013	High
		*pistilliger*	AK	Subtidal	March 2014	High
		*herzensteini*	HK	Subtidal	April 2014	High
					Janurary 2015	High
		*intermedius*	HK	Subtidal	April 2014	High
					March 2015	High
			SD		February 2014	Low
	*Icelus*	*mororane*	HK	Subtidal	March 2015	High
	*Myoxocephalus*	*polyacanthocephalus*	AK	Subtidal	March 2014	High
		*stelleri*	HK	Subtidal	November 2014	None
					Janurary 2015	Low
					February 2015	None
					March 2015	High
					April 2015	Low
		*brandtii*	HK	Subtidal	Janurary 2015	None
					February 2015	None
					March 2015	High
					April 2015	High
	*Oligocottus*	*snyderi*	AK	Tidal	March 2014	None
		*maculosus*	AK	Tidal	March 2014	None
	*Ocynectes*	*maschalis*	IZ	Tidal	April 2015	None
	*Porocottus*	*allisi*	HK	Subtidal	March 2015	High
	*Pseudoblennius*	*cottoides*	SD	Subtidal	November 2013	None
					February 2014	None
			MT		December 2013	None
	*Radulinopsis*	*taranetzi*	HK	Subtidal	April 2014	None
		*derjavini*	HK	Subtidal	April 2014	High
	*Triglops*	*pingelii*	ArO	Subtidal	July 2013	High
	*Vellitor*	*centropomus*	SD	Subtidal	November 2013	None
					February 2014	Low
Rhamphocottidae	*Rhamphocottus*	*richardsonii*	HK	Subtidal	April 2014	None
Agonidae	*Aspidophoroides*	*olrikii*	ArO	Subtidal	July 2013	High
	*Blepsias*	*cirrhosus*	HK	Subtidal	April 2014	High
	*Hemilepidotus*	*hemilepidotus*	AK	Subtidal	March 2014	None
		*jordani*	AK	Subtidal	March 2014	Low
3 families	20 genera	32 species				

## Supplementary Materials

The following are available online at https://www.mdpi.com/2218-273X/9/4/139/s1, Table S1: Accession number for molecular phylogenetic analysis.
