# Supplementary material for: Freeze Tolerance in Sculpins (Pisces; Cottoidea) Inhabiting North Pacific and Arctic Oceans: Antifreeze Activity and Gene Sequences of the Antifreeze Protein
Source: Biomolecules. 2019 Apr 6;9(4):139. doi: 10.3390/biom9040139 (PMC6523315; doi:10.3390/biom9040139)
Supplement: Supplementary file 1 [file biomolecules-09-00139-s001.pdf]

Table S1. Accession number.

Genus	Species	Accession no. 12-16S	Cytb	COI	RAG1
Rhamphocottus	richardsoni	LC126168	LC125721	LC125638	LC125804
Blepsias	bilobus	LC126246	LC125799	LC125716	LC125882
Hemitripterus	villosus	LC126241	LC125794	LC125711	LC125877
Anoplagonus	occidentalis	LC126220	LC125773	LC125690	LC125856
Aspidophoroides	monopterygius	LC126221	LC125774	LC125691	LC125857
Occella	dodecaedron	LC126219	LC125772	LC125689	LC125855
Odontopyxis	trispinosa	LC126217	LC125770	LC125687	LC125853
Pallasina	barbata	LC126218	LC125771	LC125688	LC125854
Podothecus	accipenserinus	LC126216	LC125769	LC125686	LC125852
Ulcina	olriki	LC126239	LC125792	LC125709	LC125875
Dasycottus	setiger	LC126214	LC125767	LC125684	LC125850
Ebinania	vermiculata	LC126215	LC125768	LC125685	LC125851
Eurymen	gyrinus	LC126213	LC125766	LC125683	LC125849
Psychrolutes	macrocephalus	LC126236	LC125789	LC125706	LC125872
Jordania	zonope	LC126244	LC125797	LC125714	LC125880
Scorpaenichthys	marmoratus	LC126201	LC125754	LC125671	LC125837
Hemilepidotus	hemilepidotus	LC126171	LC125724	LC125641	LC125807
Argyrocottus	zanderi	LC126187	LC125740	LC125657	LC125823
Enophrys	bison	LC126245	LC125798	LC125715	LC125881
Microcottus	sellaris	LC126197	LC125750	LC125667	LC125833
Myoxocephalus	stelleri	LC126228	LC125781	LC125698	LC125864
Porocottus	allisi	LC126227	LC125780	LC125697	LC125863
Gymnocanthus	herzensteini	LC126190	LC125743	LC125660	LC125826
Triglops	jordania	LC126170	LC125723	LC125640	LC125806
Artediellus	neyelovi	LC126184	LC125737	LC125654	LC125820
Artedius	harringtoni	LC126183	LC125736	LC125653	LC125819
Chitonotus	pugetensis	LC126186	LC125739	LC125656	LC125822
Clinocottus	analis	LC126200	LC125753	LC125670	LC125836
Oligocottus	snyderi	LC126198	LC125751	LC125668	LC125834
Icelus	sekii	LC126175	LC125728	LC125645	LC125811
Ricuzenius	pinetorum	LC126182	LC125735	LC125652	LC125818
Stelgistrum	concinnum	LC126174	LC125727	LC125644	LC125810
Icelinus	japonicus	LC126178	LC125731	LC125648	LC125814
Ascelichthys	rhodorus	LC126185	LC125738	LC125655	LC125821
Alcichthys	alcicornis	LC126202	LC125755	LC125672	LC125838
Bero	elegans	LC126242	LC125795	LC125712	LC125878
Furcina	osimae	LC126204	LC125757	LC125674	LC125840
Ocynectes	modestus	LC126237	LC125790	LC125707	LC125873
Pseudoblennius	cottoides	LC126206	LC125759	LC125676	LC125842
Vellitor	centropomus	LC126210	LC125763	LC125680	LC125846

Figure S1. Nucleotide sequences of type I AFPs from three sculpins.

```
                                                                                      [ 78]
Gt skin 1  ---------------------------------cgagctaaacaaaagtgagaATGGAC---------GCACCAGCAA
Hj skin 1  gaagttgttgatctttctcttttccaaacgcac....a.....................---------......T...
Pa skin 5  ------------------------------cac.........................TCAAATGTCA........G.
                                                                                      [156]
Gt skin 1  GAGCAGCGGCAAAGACGGCGGCGGACGCGAAGGCTGCGGCGGCCAAGACGGCGGCGGACGCTGCTAAGGCGGCGGCCA
Hj skin 1  .........................T..C...........T.AG.....T........T..GTTGGCT......AAG.
Pa skin 5  ....C....................T..A......CTA....AG.........AG...T..AAAGGCTCTA....AG.
                                                                                      [234]
Gt skin 1  AGACGGCTGCTAAA------------------------------------------------------------TAAt
Hj skin 1  .......G..GG.C------------------------------GCTGCAAGAGCTTAGtctccgtagagcggt.gcc
Pa skin 5  .......G..GG.TGCAAAAACTGCGGCGGCGGCGGTTGCAGCAGCTGCTAGAGCTTAGtctccgtagagcagt.gtc
                                                                                      [312]
Gt skin 1  tggcaaacatagttaatttgttcagtaatgcataa------------attacactataaggttcttttagggtgtgtg
Hj skin 1  ..ctt.ta..gc.cg..a...ggcaac.aa....gttaatttgttcag.a.tg.aca.gttacac.a..a...tct.t
Pa skin 5  ..cat.ta...c.cg..a...ggcaac.aa....gttaatttgttcag.a.tg..ca.gttacac.a..a...tct.t
                                                                                      [390]
Gt skin 1  tgtagggggtgggggt-atggtttgtctatgcttaattcttaatccctgatgttgctgacccaactca----------
Hj skin 1  .a---.c..g....tgg.g..ggg...c.....a.t................................----------
Pa skin 5  .agg....ag...c------------------....................................gtgtggtgct
                                                                                      [468]
Gt skin 1  ------------------------------------------------------------------------------
Hj skin 1  ------------------------------------------------------------------------------
Pa skin 5  ggttcatgtagctgaatagcacaggttattaaaacgtaaattcaagtatggaacacacatgttattttgttttgagaa
                                                                                      [546]
Gt skin 1  ------------------------------------------------------------------------------
Hj skin 1  ------------------------------------------------------------------------------
Pa skin 5  acacatgcaatcaaacaaaaaacatcttgagacgttcctgttgtgaatcagtttaatcaatttagatcaatttagtgg
                                                                                      [624]
Gt skin 1  ------------------------------------------------------------------------------
Hj skin 1  ------------------------------------------------------------------------------
Pa skin 5  ttaaaaacctgctgcttagatctcataaccaataatttgttttacagcccgggaaaagtgaaaaacctccacaaagat
                                            [656]
Gt skin 1  --------------------------------
Hj skin 1  --------------------------------
Pa skin 5  ctttctttggagctaatgtagtagcttaaaga
```
